# Supplementary material for: Searching for pneumothorax in x-ray images using autoencoded deep features
Source: Sci Rep. 2021 May 10;11:9817. doi: 10.1038/s41598-021-89194-4 (PMC8111019; doi:10.1038/s41598-021-89194-4)
Supplement: Supplementary file 1 — Supplementary Information. [file 41598_2021_89194_MOESM1_ESM.docx]

# Appendix

**Table I.** A comparison of different network architectures on a subset of CheXpert dataset[19](#_bookmark29), taken from [[49](#_bookmark59)].

| Network | pooled  AUROC | Parameters  (millions) |
| --- | --- | --- |
| AlexNet | 83.9 | 61 |
| Inception-v4 | 88.1 | 43 |
| DenseNet-121 | 86.9 | 8 |
| DenseNet-161 | 88.1 | 29 |
| DenseNet-169 | 87.6 | 14 |
| DenseNet-201 | 87.4 | 20 |
| ResNet-18 | 86.8 | 12 |
| ResNet-34 | 87.2 | 22 |
| ResNet-50 | 88.1 | 26 |
| ResNet-101 | 87.6 | 45 |
| ResNet-152 | 88.2 | 60 |
| VGG-13 | 85.0 | 133 |
| VGG-16 | 85.6 | 138 |
| VGG-19 | 85.7 | 144 |

**Table II.** A comparison of different network architectures on the COVID-CT dataset[50](#_bookmark60), taken from [[51](#_bookmark61)]

| Network | Accuracy (%) | F1 score | AUC | Parameters  (millions) |
| --- | --- | --- | --- | --- |
| AlexNet | 86*.*85 *±* 13*.*66  93*.*83 *±* 6*.*97  95*.*44 *±* 8*.*02  93*.*62 *±* 6*.*17  93*.*29 *±* 5*.*69  89*.*26 *±* 8*.*80  90*.*16 *±* 7*.*72  96*.*20 *±* 4*.*95  95*.*97 *±* 7*.*18 | 0*.*85 *±* 0*.*16  0*.*94 *±* 0*.*06  0*.*96 *±* 0*.*07  0*.*94 *±* 0*.*06  0*.*94 *±* 0*.*05  0*.*90 *±* 0*.*08  0*.*90 *±* 0*.*08  0*.*96 *±* 0*.*05  0*.*96 *±* 0*.*07 | 0*.*94 *±* 0*.*04  0*.*96 *±* 0*.*04  0*.*98 *±* 0*.*03  0*.*98 *±* 0*.*02  0*.*98 *±* 0*.*02  0*.*96 *±* 0*.*03  0*.*97 *±* 0*.*03  0*.*98 *±* 0*.*03  0*.*97 *±* 0*.*05 | 61 |
| GoogLeNet |  |  |  | 7 |
| ResNet-18 |  |  |  | 12 |
| ResNet-50 |  |  |  | 26 |
| ResNet-101 |  |  |  | 45 |
| VGG-16 |  |  |  | 138 |
| VGG-19 |  |  |  | 144 |
| DenseNet-201 |  |  |  | 20 |
| MobileNet-v2 |  |  |  | 3.5 |

A recent paper[49](#_bookmark59) has compared the performance of different deep neural network architectures for chest x-ray classification. Different variations of five network architectures including ResNet, DenseNet, VGG, SqueezeNet, Inception v4 and AlexNet were trained for 8 epochs on a subset of the CheXpert dataset[19](#_bookmark29). The used dataset was created by choosing only the frontal images of Cardiomegaly, Edema, Consolidation, Atelectasis and Pleural Effusion labels of the CheXpert dataset where the images with uncertain labels were excluded. Table [I](#_bookmark63) is taken from Table 1 of [[49](#_bookmark59)] with some row exclusions and column additions. As it can be seen in Table [I](#_bookmark63), ResNet-152, Inception-v4, DenseNet-161 and ResNet-50 have shown the best performances of 88.2, 88.1, 88.1 and 88.1 with 60, 43, 29 and 26 million parameters, respectively. On the other hand, AlexNet and all of the VGG versions (VGG-13, VGG-16, VGG-19) have had the lowest pooled AUROCs of 83.9, 85.0, 85.6 and 85.7 while they have relatively large numbers of parameters, 61, 133, 138 and 144 million, respectively. DenseNet-121 has been able to yield high quality results, 86.9, while utilizing much less parameters compared to other networks, only 8 millions, and consequently making the training time and further analysis of the results much easier. These observations suggest DenseNet-121 as an efficient architecture for working with chest x-ray images. Another recent study[51](#_bookmark61), compares the performance of 16 different network architectures on the classification of COVID-19 using CT scan images. The dataset used for this study, namely COVID-CT-Dataset, contains 349 CT images of 216 patients diagnosed positive for COVID-19 and 397 CT images of non-COVID-19 subjects[50](#_bookmark60). The performance of each network is measured using different metrics such as Accuracy, Sensitivity, Specificity, F1-score, and AUC after 6 epochs of training. The results, shown in Table [II](#_bookmark64) extracted from Table 2 of [[51](#_bookmark61)], indicate that DenseNet-201 has the best accuracy, F1-score and AUC while AlexNet, VGG-16 and VGG-19 have had the lowest performance with regards to all of the investigated metrics. This confirms DenseNet being a suitable architecture for medical image analysis.
